# A Systematic Review of Artificial Intelligence Applications Used for Inherited Retinal Disease Management

**DOI:** 10.3390/medicina58040504

**Published:** 2022-03-31

**Authors:** Meltem Esengönül, Ana Marta, João Beirão, Ivan Miguel Pires, António Cunha

**Affiliations:** 1 Escola de Ciências e Tecnologia, University of Trás-os-Montes e Alto Douro, Quinta de Prados, 5001-801 Vila Real, Portugal; meltem.esengonul@gmail.com (M.E.); impires@it.ubi.pt (I.M.P.); 2 Department of Life Sciences Engineering, University of Applied Sciences Technikum Wien, 1200 Vienna, Austria; 3 Department of Ophthalmology, Porto University Hospital Center, 4099-001 Porto, Portugal; analuisamarta2@gmail.com (A.M.); brandaobeirao@gmail.com (J.B.); 4 Abel Salazar Biomedical Sciences Institute, University of Porto, 4050-313 Porto, Portugal; 5 Instituto de Telecomunicações, Universidade da Beira Interior, 6200-001 Covilhã, Portugal; 6 Instituto de Engenharia de Sistemas e Computadores, Tecnologia e Ciência, 3200-465 Porto, Portugal

**Keywords:** inherited retinal disease, artificial intelligence, machine learning, deep learning, systematic review

## Abstract

Nowadays, Artificial Intelligence (AI) and its subfields, Machine Learning (ML) and Deep Learning (DL), are used for a variety of medical applications. It can help clinicians track the patient’s illness cycle, assist with diagnosis, and offer appropriate therapy alternatives. Each approach employed may address one or more AI problems, such as segmentation, prediction, recognition, classification, and regression. However, the amount of AI-featured research on Inherited Retinal Diseases (IRDs) is currently limited. Thus, this study aims to examine artificial intelligence approaches used in managing Inherited Retinal Disorders, from diagnosis to treatment. A total of 20,906 articles were identified using the Natural Language Processing (NLP) method from the IEEE Xplore, Springer, Elsevier, MDPI, and PubMed databases, and papers submitted from 2010 to 30 October 2021 are included in this systematic review. The resultant study demonstrates the AI approaches utilized on images from different IRD patient categories and the most utilized AI architectures and models with their imaging modalities, identifying the main benefits and challenges of using such methods.

## 1. Introduction

Inherited Retinal Diseases (IRDs) are hereditary illnesses that impact 1 in every 3000 people globally. They can be characterized by dominant, recessive, or X-linked mutations caused by Mendelian defects in the 280 identified genes thus far [1,2]. According to a recent analysis of 10,044 mutations by studying changes in 187 IRD-associated genes, researchers have calculated that 2.7 billion people worldwide are carriers of an IRD disease-causing mutation, with 5.5 million predicted to be afflicted [3]. There are several types of IRDs, such as Retinitis Pigmentosa (RP), Choroideremia, Stargardt Disease (STGD), Cone-Rod Dystrophy (CRD), Leber Congenital Amaurosis, etc. One of the most common types of IRD is Retinitis Pigmentosa, which can be distinguished by a gradual, cumulative degeneration of photoreceptors, most notably rods, usually accompanied by cone photoreceptor degeneration [4,5]. It is stated that early RP decreases night and peripheral vision, although central vision stays unchanged until subsequently in the disease [4].

On the other hand, Choroideremia is an X-linked hereditary retinal disease that causes peripheral visual field reduction while preserving the field of vision in the central retina [6,7]. Patients with Choroideremia have night blindness and peripheral visual field loss progress over 3–5 decades. Most patients maintain good visual acuity until a central island of foveal vision is lost. The prevalence rate of Stargardt Disease (STGD) is estimated at around 1 in 10,000, which converts it into the most common type of juvenile-onset macular degeneration [8,9]. When clearance is disrupted, lipofuscin accumulates prematurely in retinal pigment epithelial (RPE) cells, which is a characteristic of the illness and is considered to cause toxicity and, eventually, photoreceptor cell death [8].

There are several techniques used in the detection of IRDs. Ophthalmologists determine first the visual function by identifying two main characteristics: refraction and visual acuity [10]. They often assess visual fields (or perimeter) and color vision, usually altered in these patients. This might have implications for legal purposes (e.g., driving license). Besides these examinations, Electroretinography (ERG) and Electrooculography (EOG) can still be practical in the field [11,12]. The most widely used technique is retinal imaging, which aids in diagnosing retinal disorders such as minimally observable alterations that cannot be identified with fundus examination alone [13,14]. Thus, the use of retinal imaging surpassed the retina physiological exams. The two most important technologies used in the detection of IRD are high-resolution optical coherence tomography (OCT) and fundus autofluorescence (FAF) [15,16]. The OCT approach has allowed the detection of distinct patterns of posterior pole changes and involvement of peripapillary retinal nerve fiber layer (RNFL) thickness in IRD patients [17,18,19,20]. In FAF, on the other hand, the retina’s inherent fluorescent material is seen, which is mostly lipofuscin [21]. Wide-field research revealed a link between aberrant FAF regions and visual function in RP and CRD [21]. In recent years, there have been several advancements in the treatment of inherited retinal disorders, which include drug therapies like neuroprotectants, visual cycle modulators, anti-inflammatory drugs, and antioxidants [22] and gene therapies via gene replacement and gene editing, such as Adeno-Associated Virus (AAV) mediated Luxturna, as well as optogenetics [23].

Artificial Intelligence (AI) technology is gaining popularity with retinal imaging due to enhanced processing power, massive data, and novel algorithms [24]. Machine Learning (ML) is a branch of AI that seeks to answer the challenge of creating machines that learn independently [25]. Whereas Deep Learning (DL), a subset of ML, enables computational models of numerous processing layers to learn data representations with varying degrees of abstraction [26]. AI and its subfields can be used for several biomedical applications. It can assist physicians in following the patient disease cycle, help them with diagnosis, and show suitable treatment options. Each method used may answer one or more specific AI problem types, such as segmentation, prediction, recognition, classification, and regression. However, the number of AI featured studies related to IRDs is still only a few. Thus, this study aims to review artificial intelligence techniques applied to Inherited Retinal Disease management from diagnosis to treatment. The review includes papers submitted until 30 October 2021.

The novelty of this paper compared to previous systematic reviews on the subject is that although several studies are investigating the use of AI in ophthalmology, many of them include diseases such as Diabetic Retinopathy, Glaucoma, Age-related Macular Degeneration, and others. This paper focuses on specifically Inherited Retinal Diseases and broadens the spectrum of ophthalmologic use of AI. It also uses a Natural Language Processing (NLP) based reviewing technique to determine the AI methods applied to IRD images, the most common architectures, and the imaging modalities used. In other words, a type of AI is used as a tool to detect AI methods used for IRD management, which is also a newer methodology in the field. The occurrences of several properties are identified with statistical analysis methods, relative graphs are derived, and the most relevant keywords are correlated with occurrence mapping. The implication of using such technologies in the medical field is highlighted with advantages and disadvantages. In Section 2, materials and methods are described in more detail, including the research questions, inclusion criteria, exclusion criteria, search strategy, and extraction of study characteristics. The search results are then reported in Section 3, where the 11 included articles are extensively evaluated. In Section 4, an examination and summary of the findings can be seen, and in Section 5, the study’s conclusion and future perspective are indicated.

## 2. Materials and Methods

### 2.1. Research Questions

In this study, five essential research questions were considered, which are the following:

- Which artificial intelligence, machine learning, and deep learning methods are used for IRD management? (RQ1);
- In which way can these methods improve IRD detection? (RQ2);
- Which algorithms/architectures are most used in IRD management? (RQ3);
- What are the complications of using these methods for IRD detection? (RQ4);
- What imaging modalities are used along with these methods for different types of IRD identification? (RQ5).

### 2.2. Inclusion Criteria

This study explores the association of artificial intelligence, machine learning, and deep learning methods with Inherited Retinal Disease Management. For this systematic review, the inclusion criteria consist of studies that:

- are published in the English language;
- are published between the years 2010 and 2021;
- investigate Inherited Retinal Diseases or their subtypes;
- use at least one of the artificial intelligence, machine learning, or deep learning techniques;
- are addressing the five main artificial intelligence problems (Segmentation, Prediction, Recognition, Classification, and Regression);
- are not categorized as either survey or review;
- conform to the “Explainability” rule of artificial intelligence.

### 2.3. Exclusion Criteria

On the other hand, the exclusion criteria used in this systematic review are reports that:

- are not published in the English language;
- are published before 2010 and after the completion of the study (30 October 2021);
- do not clearly state the purpose, dataset, patient distribution, methods, and conclusion of the study;
- are not human studies;
- do not investigate Inherited Retinal Diseases or their subtypes;
- do not use any artificial intelligence, machine learning, or deep learning techniques;
- are categorized as either survey or review;
- do not conform to the “Explainability” rule of artificial intelligence.

### 2.4. Search Strategy

Several electronic databases were utilized to identify papers that conform to the inclusion and exclusion criteria in this systematic review. These databases are IEEE Xplore, Springer, Elsevier, MDPI, and PubMed, using a novel natural language processing (NLP) approach. This method automates the process of scanning scientific papers and trend analysis meta-studies [27]. It provides a rigorous and complete qualification and relevancy assessment of publications by employing NLP, allowing the user to refocus on reviewing a smaller range of potentially related papers [27]. This systematic review was conducted using the following research terms: “Inherited retinal disorder” AND “Inherited retinal disease” OR “Fundus autofluorescence”. This systematic review aims to illuminate artificial intelligence-associated methods used for Inherited Retinal Disease management from diagnosis to treatment. The study was conducted with all authors who reviewed each paper individually to investigate the conformity of the paper to the inclusion criteria unanimously and the exclusion criteria. This study was completed on 30 October 2021.

### 2.5. Extraction of Study Characteristics

The study analysis results show that there are various critical pinpoints associated with IRD management and AI methods, such as disease type, imaging modality, dataset size, patient distribution, the purpose of the study, identified AI problem type(s), applied algorithms, architectures of the algorithms, and their best performance results. The resulting chart can be seen below in Table 1.

### 2.6. Statistical Analysis Methods

After selecting the studies for the meta-analysis, each property occurrence, such as the disease type, patient distribution cohort numbers, imaging modality, dataset size category, identified AI problem types, algorithms, and models, were counted. The results are given in the following Section 3.

Several graphs were obtained using the statistical analysis methods of the total valid reports to evaluate the frequency of common keywords, different IRD disease types, AI problems, and affected eye parts. Using the pivot analysis with special keywords, the occurrences were counted regarding several properties such as but not limited to: Year, Source, Study Type, Disease Type, Affected Eye Parts, Symptoms, General AI Topics, AI problem, AI Algorithms, and Explainability rule. Relevant graphs were later presented in Section 4, Discussion, including the keyword occurrence map and the distribution graphs of several properties.

## 3. Results

**Figure 1:**
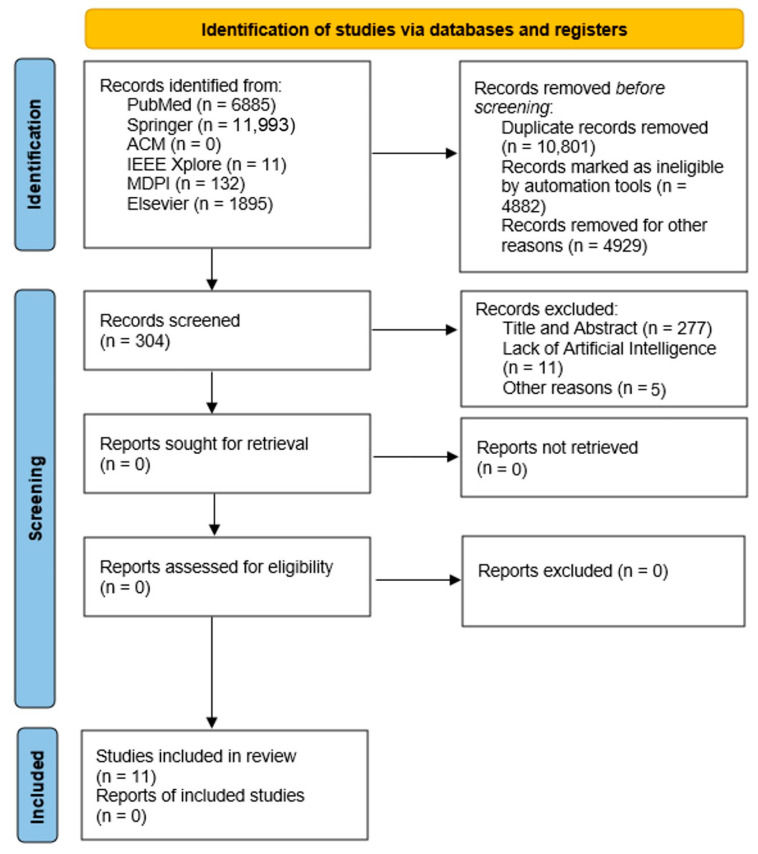
Flow Diagram.

The following Figure 1 represents the Flow diagram adapted from the study by Page et al. [39]. A total of 20,906 articles were identified using the NLP method from the databases mentioned above. There were no other additional registers considered in this study. By analyzing the articles, it was found that 10,801 of them were duplicates, so they were removed before the screening. For the screening process, there were 10,105 articles included. The screening process removed 4835 due to incomplete data and 47 because of invalid years. Thus, 4882 records were excluded in the first screening process by evaluating the title/abstract. A total of 5223 papers were assessed for eligibility, and 4919 were not included due to not conforming to inclusion criteria six, indicating that articles were either survey or review types. Later, 293 were excluded due to the lack of artificial intelligence, machine learning, or deep learning methods. Finally, 11 studies are included in the review as part of the qualitative and quantitative analyses.

Using the meta-data analyses, necessary information is gathered. This research consists of articles published from 2010 to the search’s completion date, 30 October 2021. Table 1 depicts the distribution of the article selection and provides a relevant comparison regarding the types related to the specific properties extracted from the articles. Most of the articles included were from 2020 with a 46% rate, followed by 2018 with a 27% rate. As shown in Table 1, eight different disease types were associated with the included articles, such as Retinitis Pigmentosa, Stargardt Disease, Chloridemia, Macular Dystrophy, Best Disease, Blue Cone Monochromacy, Geographic Atrophy, and Pseudo-Stargardt Pattern Dystrophy. However, in some cases, more than one type of disease was related to the study; thus, the number of occurrences was counted to reveal which one was used the most. Retinitis Pigmentosa and Stargardt Disease shared the rate with 29% representing five occurrences each, followed by Chloridemia with a 12 percent rate. The remaining categories occurred once, each representing a 6% percentile with 30% of the whole. When it comes to IRD screening, the imaging modality used to capture the images is also crucial. As a result of the analyses depicted in Table 1, six different modalities were identified for IRD screening: Optical Coherence Tomography, Adaptive Optics Scanning Light Ophthalmoscope, Spectral Domain Optical Coherence Tomography, Fundus Autofluorescence, Pupillometer, and Fundus Photography. The most used modality appears to be Optical Coherence Tomography with an occurrence rate of 37%, followed by Fundus Autofluorescence with 27%. All the articles mentioned in Table 1 dealt with small datasets with image numbers below 2000. Small dataset use can affect the outcome widely in many cases, and in fact, some authors mention difficulties using such images.

Nonetheless, they are a considerable contribution to the field. If we were to categorize dataset size ranges into four categories with an image number between 0–100, 101–500, 501–1000, and over 1000 images, we can see a clear distinction that most of them fell into the category of 0–100 with a percentage of 46%. It may also be argued that these were mostly related to image segmentation. In these cases, patches may be used, thus featuring fewer data. Still, we also need to look at the distribution of patient numbers. Suppose we use a similar range approach to the dataset categorization. In that case, we can see that again, most of the patient data size fell into the first category with a patient number between 0–100 with a value of 73%. Although here, one of the studies does not specify the number of patients but only the number of eyes. Due to the varying availability of patients, studies also show different numbers of cohorts utilized to perform the IRD screening methodologies. The dataset’s variability can sometimes be beneficial or may cause other issues. We can categorize these into four main types: studies using one cohort, two cohorts, three cohorts, and four cohorts. With the most significant majority of 73%, studies favored using two cohorts featuring mostly one healthy group and another with a specific disease which is the minimum to apply image classification algorithms.

A total of 7 different algorithms are distinguished from the methodologies. These algorithms included CNNs, MDRNNs, Ensemble Classifiers, DNNs, Feature Extraction, SVMs, and DCNNs. Most used algorithms were identified as CNNs with a 42% rate. Several models were chosen for the application featuring 12 distinct model types for these algorithms. For the CNNs, Inception V-3 and Resnet101 appeared to be used slightly more than others, with a 13% occurrence rate each. It is difficult to compare each performance of the models as they are based on different datasets with different sizes and different imaging modalities and algorithms. However, it can be said that most similar AUC-ROC scores were achieved in the studies both using Resnet101 from [34,38], although they were applied to varying cohorts.

The study [28] uses a CNN-based algorithm with a MatConvNet architecture to automatically segment the IRD types such as Retinitis Pigmentosa and Choroideremia from OCT images. The method used in the article follows a 5-step approach: preprocessing, manual grading, patch extraction, neural network training using patches, and postprocessing. They train the CNN algorithm using B-scan patches encompassing portions of the Ellipsoid Zone (EZ), marked depending on the occurrence of en face images at the patch’s central A-line point. The authors mention that in comparison to manual segmentation by a professional, their process, the further bimodal thresholding of probability maps using an Otsu scheme and morphological procedures generating binary maps of the segmented preserved photoreceptor regions, provides good accuracy results. In total, 81,600 patches of B-scans obtained from 20 Chloridemia and 22 Retinitis Pigmentosa patients apply to the segmentation and classification problem of IRD, namely for the measurement of preserved Ellipsoid Zone loss. To evaluate the performance of the used method Jaccard Similarity Score (JSS) was measured. For automatic segmentation of Retinitis Pigmentosa, the JSS value was assessed as 0.894 ± 0.102 instead of the best performance value of 0.912 ± 0.055 for Chloridemia compared to manual grading. Here, the main issue faced seems to be the lack of inter-subject illustration. As the number of subjects is low, the features extracted pose a poor representation of the whole population of the diseased. Authors add that the IRD type variation is lacking as well. They propose in the future to operate along with other institutions to gain access to more extensive databases to avoid the problem. Overall, this study demonstrates that it is possible to use an automated algorithm to segment and classify the preserved effectively and disrupted EZ areas in Choroideremia and Retinitis Pigmentosa and even other types of IRD with further research from OCT images.

The authors of [29] apply a robust automated cone identification technique based on a Multi-Dimensional Recurrent Neural Network (MDRNN) on images from an Adaptive Optics Scanning Light Ophthalmoscope (AOSLO) to identify cone photoreceptors in both healthy subjects and Stargardt Disease subjects. For this segmentation and classification of problem-based network architecture, the authors use a combination of convolutional layers, MDLSTM layers, and fully connected layers to find the best model with a Dice score of 0.9577 for the validation set. There were 290 images used for this study from 8 Stargardt Disease patients and 17 healthy subjects. Authors depict that the main challenge with utilizing gradient-based learning algorithms from AOSLO split detection images is discovering local optima and categorizing everything as background. To avoid this issue and overcome the imbalanced classes, Generalized Dice Loss (GDL) is used in this study. Another significant issue is the necessity of deeper investigation before the extensive application of such methodologies. Authors add that to guarantee strong performance, it is critical to carry out a systematic clinical assessment of the approach, taking into account a wider variety of diseases and AOSLO imaging instruments. Nevertheless, this study can aid the detection of the location of the cone structures both in healthy and unhealthy subjects with Stargardt Disease in a fast and robust manner.

In [30], the authors also focus on the Ellipsoid Zone segmentation, like in the study [29], in which they investigate 16 eyes of patients with Choroideremia with a machine learning algorithm based on random forests that were created to automatically recognize continuous patches of maintained ellipsoid zone structures from OCT images. The authors use 20 volumetric scans obtained from the nine Choroideremia and five healthy subjects to train the random forest classifier. They achieve, before post-processing, a JSS value of 0.845 ± 0.089 when compared to a value of 0.876 ± 0.066 after post-processing. Twelve characteristics based on maximum reflectance, minimum reflectance, and minimum reflectance position projections were employed for categorization. The authors mention that the brightness in the images created by reflectance value projections and the blackness in the images generated by minimum position projection were the significant features that aided in identifying either partially or entirely preserved EZ regions. Thus, one major challenge of the study is the shadows created by big vessels in the inner retina, vitreous floaters, or pupil color fringing. However, the authors point out that using Gaussian filters with gradually more significant kernels for projections of maximum reflectance and minimum reflectance may reduce the errors associated with shadows.

In [31], a Deep Neural Network method used Medic Mind, an online deep learning platform that uses the Inception V-3 pre-trained model, for the identification of the genetic marker of both Inherited Retinal Disease (IRD) types: Macular Dystrophy induced by ABCA4 and RP1L1 gene mutations, Retinitis Pigmentosa induced by EYS gene mutations as compared to healthy individuals is demonstrated. In total, 178 images were obtained from 75 individuals with ten patients with the ABCA4 gene, 20 patients with the RP1L1 gene, 28 patients with the EYS gene, and 17 healthy subjects using horizontal, vertical cross-sectional scans spectral-domain optical coherence tomography (SD-OCT) were used. The mean training accuracy ranged from 90.6 to 100.0 percent for the classification problem, whereas testing accuracy was 90.9 percent (82.0–97.6). Furthermore, the classification performance accuracy per gene category was 100% for ABCA4, 78.0% for RP1L1, 89.8% for EYS, and 93.4 percent for Healthy. The study identifies the main challenge as the lack of more extensive datasets withholding rare types of IRDs and difficulty in identifying identical structural alterations within the same cohort. As a solution, the authors suggest using more extensive mechanism groups/cascades and various morphologies as part of the training set.

In [32], a deep learning-based technique to segment hyper autofluorescence visible flecks from FAF images to measure structural aspects of Stargardt Disease is implemented. The authors use a CNN approach with encoder and decoder parts for this segmentation problem. A ResNet-34 pre-trained model is used for down-sampling whereas, for up-sampling, a U-Net pre-trained model is used. There are 47 images used for this ResNet-UNet model, consisting of 31 patients showing definite, well-defined so-called pisciform (fishbone) pattern lesions, whereas 16 patients had sparsely speckled lesions with no pisciform lesions. Before training the dataset, the authors applied the Contrast Limited Adaptive Histogram Equalization (CLAHE) method to pre-process the images and augment the pictures using rotation, inversion, and magnification techniques. For the performance measurement, Dice Loss Score between manual and automatic segmentation is quantified, and 0.8 is the best result achieved in this measurement for subject ID 10 with a discrete fleck type. Due to the rarity of these Stargardt patients (STGD1), a minimal number of images were utilized. To avoid this limitation, they adopted a patch-based strategy. However, their training set still included eyes with few flecks and speckled FAF signals; in the future, the authors suggest not utilizing these images instead of applying the algorithm to a dataset with the maximum number of well-defined pisciform fleck lesions. They also find that CLAHE creates a constraint in the study as it changes the look of the hyperautofluorescent specks instead of manual segmentation from the raw images. For further studies, it is advised to either segment FAF images after CLAHE manually or investigate alternative image modification techniques, such as the area erosion approach, to preserve the look of the hyperautofluorescent flecks.

The authors of [33] use a chromatic pupillometer to detect Retinitis Pigmentosa amongst pediatric patients by employing a machine learning-based Clinical Decision Support System (CDSS). The characteristics retrieved from the pupillometric data are classified using two separate Support Vector Machines (SVMs), one for each eye. In this study, the authors can achieve an accuracy value of 0.846, a sensitivity of 0.937, and a specificity of 0.786, which are promising for future practice. Importing raw data and pupillary diameter data pre-processing, feature extraction of pupillary data and reduction, optimization of hyperparameter, and ultimately training the supervised classifier are the primary phases of utilizing the classifier. Out of the 38 chromatic pupillometry data, only 30 were used due to signal interference used for this classification problem.

One advantage of this method of data extraction mentioned by the authors is that in comparison to traditional diagnostic procedures, notably electrorheological tests, no electrodes are required on the patient’s body in this situation: this seems to be especially useful when working with younger individuals. As well as this, the duration of the examination is relatively shorter than the traditional electroretinogram. Still, some issues seem to be faced during the application of the process. Due to the limited quantity of data supplied for this study, the authors point out that more experiments with a more significant data source are needed to test the overall system performance by other devices. The typical appearance of movement artifacts emerged as an important issue during the data collection stage. Authors suggest studying other systems with various frames and technologies based on smartphones.

In [34], several types of IRD, such as Retinitis Pigmentosa (RP), Best Disease (BD), and Stargardt Disease (STGD), are investigated. For this classification problem of subtypes of IRD, they utilize a multilayer deep convolutional neural network (CNN) based on a pre-trained ResNet101 model from FAF images. A total of 483 FAF images were used to train and validate the DCNN. The FAF images from patients are 73 from healthy subjects and 410 from subjects with IRDs: 125 from STGD patients, 160 from RP patients, and 125 from BD patients. Authors also apply augmentation techniques for dealing with a small dataset and an Adam optimizer. The system achieved a ROC-AUC of 0.998 and a PRC-AUC of 0.986 for STGD, as per RP ROC-AUC evaluated at 0.999 and PRC AUC at 0.999 being the best performance.

On the other hand, BD performance results in ROC-AUC of 0.995 and PRC AUC of 0.988, similar to healthy controls were with ROC-AUC of 0.998 and the PRC-AUC of 0.989. As for the accuracy, the program performs beneficiary results for further applications with the IRD classifier system with an overall accuracy score of 0.95. In this study, one of the significant drawbacks mentioned by the authors is the use of a relatively small dataset, and the dataset lacks the rarest types of IRD. It uses the most common three in which they had sufficient training data. The authors believe that the model may not perform as well when trained on different images since the variability of the dataset is due to eye-level segmentation of the dataset and the usage of a training/validation/test split. The molecular genetic testing for a portion of the included eyes seems to be also not provided. Thus, authors suspect difficulty in predicting the classification algorithm in a clinical environment due to the broad spectrum and genotypic and phenotypic heterogeneity of IRDs. Furthermore, picture noise and considerable inter-individual and intra-individual heterogeneity in media opacities, lipofuscin concentration, and genetic expression influence FAF imaging and may provide a big obstacle. To tackle these obstacles, the authors mention confidence estimates and KDE graphs to complement the work and application on a larger dataset that might be available with the collaboration of more institutions.

The authors of [35] apply two different CNN-based models to classify Stargardt Disease patients from healthy individuals. For the first approach, they use a pre-trained network based on VGG19, whereas, for the second, a custom network is used, such as LeNet from [40]. For model 1, training based on a pre-trained VGG19 framework took 50 epochs to emerge to the peak accuracy of 99.8 percent on the validation set. When the proposed method ran on different testing data, it produced findings with 99.0 percent accuracy, 96.0 percent sensitivity in moderate STGD, 99.5 percent sensitivity in severe STGD, 98.0 percent specificity, and a mean JSS of 0.958. When used as a binary classification algorithm, it produces a result of the accuracy of 99.6%, sensitivity of 99.8%, specificity of 98.0%, and a JSS of 0.990.

In comparison, to achieve over 90% accuracy on the validation set, training model 2 took over 250 epochs. In total, 749 OCT scans were obtained from 60 STGD patients and 33 healthy subjects for the classification problem from OCT images. Apart from the common issue of a small dataset, the authors also experience a topic related to the imaging modality, OCT. They mention that image noise and artifacts in OCT imaging provide a considerable problem, but deep learning helps to avoid these issues. Further, consecutive studies should embrace genetic data from enough patients to represent the enormous phenotypic and genetic variability of STGD to aid in phenotype-genotype connection.

In [36], a technique utilizing ensemble classifiers is employed, namely random forest for the prediction and segmentation problem regarding Blue Cone Monochromacy (BCM) subtype patients’ foveal vision performance measurement in a clinical study of intravitreally provided vector-gene from cross-sectional retinal composition images from OCT. They point out that this procedure only focuses on foveal cones and not extra central disrupted cones in these retinas. Patient distribution here is 26 to 16 for IRD and BCM, respectively. The dataset includes one eye examination from each patient, so there are 42 OCT scans considered. Two regression analysis methods are used for visual outcome prediction for the machine learning application. First, the machine learning-based approach used was the random forest (RF), which predicts the link between foveal function and retinal anatomy (FS-VA). There were two groups for the segmentation analysis based on the input parameter. The first group was related to foveal sensitivity measures, whereas the second group was for visual acuity measures. The second method uses non-linear regression analysis, curve fitting for the correlation between photopigment and thresholds of the visual-retinal structure obtained from psychophysics or electrophysiology. For all techniques, the root-mean-square error (RMSE) was employed as a metric of model performance. RSMEs for segmentation Model I-FS and I-VA of Random Forest techniques depicted prediction vs. evaluated values of 2.91 and 0.159, respectively. The visual acuity segmentation with the ML method is closer to the real values than the foveal sensitivity ML-based segmentation method, making it the best performance. Again, for the curve fitting technique, similar results were obtained: 2.91 for FS whereas 0.174 for VA. Like in the other studies, the small dataset seems to be the issue of the difficulty of the foveal ONL calculation manually. These can be resolved with machine learning techniques. Authors mention that diseases with vision and structures of rod-mediation residue should be focused on and investigated for further studies.

Unlike the studies above, the authors of [37] utilized color fundus photographs of 1670, of which 1153 are Retinitis Pigmentosa images, and 517 of them are images from healthy subjects to detect RP occurrence. Three different pre-trained CNN models (Inception V3, Inception Resnet V2, and Xception) were applied for classification problems with an Adam optimizer, and fine-tuning was used to enhance the robustness of the method. They found out that the Xception model performed the best, with an AUROC score of 99.46%, accuracy of 0.960, sensitivity of 0.9571, specificity of 0.9853, and F3 score of 0.9599. Compared to a general ophthalmologist and retinal specialist classification, the method proposed achieved the best results amongst all performance metrics. Grad-CAM was produced in this study using the model weights and the feature map to show the critical region in the photographs. Authors emphasize that the more the redness of the part, the more significant the AI model finds the area to relate to RP qualities. This technique is essential to aid ophthalmologists or retinal specialists when making clinical decisions. One major problem in these studies mentioned by the authors is that the system can grade images differently if they are obtained from different sources. All the pictures in this study are obtained with the same camera to avoid this. This can help immensely. However, it can also create a homogeneity issue when tested with other datasets, which leads to overfitting, as pointed out by the authors.

In [38], the focus is on the identification of retinal atrophies, especially three types such as Geographic Atrophy (GA), Stargardt Disease (STGD1), and Pseudo-Stargardt patterns Dystrophy (PSPD) from FAF images with the use of deep learning techniques. Their dataset includes 314 FAF images, of which 204 of them are jointly from STGD1 and PSPD, and the remaining 104 are from GA patients. They use DCNN based architecture with a ResNet101 pre-trained model for this classification problem. For the first method, they divide the dataset into training, validation, and testing with a 70:10:20 ratio. Later, they use the augmentation technique since the dataset is small on the training set. They utilize Adam optimizer and validate in real-time. The model’s performance measures an accuracy score of 0.921 and AUC-ROC of 0.990. For the second method, they apply K-fold cross-validation, which divides the dataset randomly into k mutually exclusive groups of equal size with a 70:30 training vs. test ratio. The test set was then divided into ten equal-sized subgroups for ten-fold cross-validation. Here, they achieve an accuracy score of 0.873 and AUC-ROC of 0.958.

In that sense, the first method slightly outperforms the second. Nevertheless, the field benefits from these applications as they can effectively classify both etiologies of atrophy. Similar to [37], this research uses integrated gradient visualization to help clinicians view why the model makes such predictions. This study investigates the distinction between STGD and PSPD posing as GA. It reveals that aside from these diseases, Cone Dystrophy, Adult Vitelliform Dystrophy, North Carolina Macular Dystrophy, Doyne Honeycomb Dystrophy, Sorsby Macular Dystrophy, X-linked Retinoschisis, and Maternally Inherited Diabetes and Deafness (MIDD) may also present as GA and create complications. Thus, further research should concentrate on a more extensive range of IRDs for GA diagnostic processes to avoid this issue. Moreover, disease prognosis is eminent when dealing with STGD, PSPD, and GA, which makes phenotyping refining vital in these cases to make an efficient assessment of the RPE atrophy and consideration of patient history ERG, and testing genetics.

In summary, by thoroughly examining the 20,906 reports about inclusion and exclusion criteria, 11 were selected for the meta-analysis according to the article’s purpose. The chosen articles use AI or its subtype methodologies and apply them to the management of IRD. Out of the selected articles, although few, the most common diseases investigated in this regard were Retinitis Pigmentosa and Stargardt Disease. The most common algorithms used for AI purposes are CNNs. The patient distribution, type of AI models used, and purpose of the studies varied immensely. Thus, it is hard to determine the most common types of these. All the studies achieved outstanding results depending on the metrics chosen. The most common metric used was Accuracy. The accuracy results are all above 84% and, in some cases, even achieve 100%. However, when looking at the dataset size, they were primarily small, accounting for most of them with 0–100 images. In that sense, accuracy should not be considered the only evaluation metric.

Findings from the study suggest that AI in IRD management is fundamentally associated with detecting the disease or its subtypes. For detecting the disease, Ellipsoid Zones segmentation or classification is highly beneficial. Phenotyping, as well as genotyping, is strictly crucial for the identification of the IRD disease, and AI methods can aid by reducing the person-hours on this matter. Specifically, cone structure localization or specific gene markers are easier to find using AI methodologies. This is both beneficial for the detection and treatment of specific IRD types. That is to say that there are still issues regarding the use of such technologies, mostly related to imaging such as image noise and movement artifacts. Some problems are also associated with the data and its distribution as data availability is scarce, and the absence of rare IRD types in the data should be considered. It is also possible that the disease can present itself as another in the imaging data. Thus, it is essential to refer to genotyping results and patient history.

## 4. Discussion

**Figure 2:**
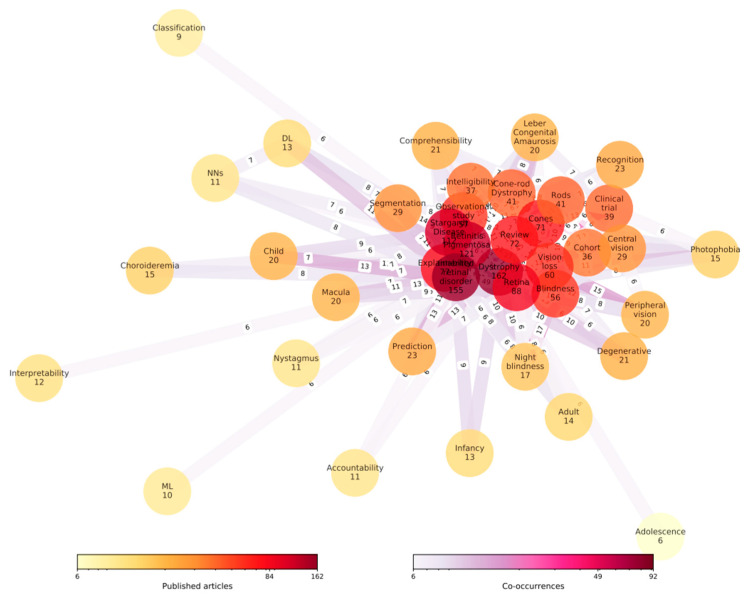
Keywords occurrence map.

In this study, Artificial Intelligence and its subfields were examined by applying images from IRD patients. Meta-analyses were performed on 11 studies reflecting the inclusion and exclusion criteria. Using an NLP-based systematic review, it was possible to identify the most general keywords and their relevance graphs to each other, which can be seen in the following Figure 2. The words with the most occurrences are shown in darker red, whereas the least occurrences are shown in yellow. However, we have removed the review papers and surveys to focus on specific studies in the later elimination stages. According to this graph shown in Figure 2, these keywords were: Inherited Retinal Disorder, Stargardt Disease, Retinitis Pigmentosa, Dystrophy, Retina and Review, and Explainability. It can also be understood that the most co-occurrence happened between Inherited Retinal Disorder and Prediction and between Dystrophy and Prediction and Inherited Retinal Disorder and Macula.

**Figure 3:**
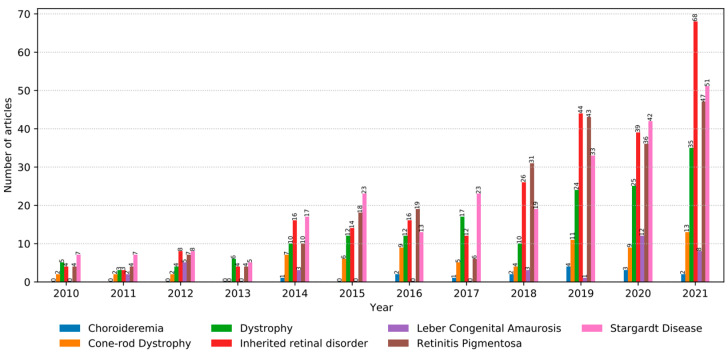
Distribution of diseases according to years.

The analyses recorded that besides the general topic, Inherited Retinal Disorder, Stargardt Disease and Retinitis Pigmentosa were the two most prominent types of inherited retinal dystrophies with 25% and 23% occurrence rates between 2010 and 2021, respectively. The distribution of all studies according to their years and diseases can be seen below in Figure 3.

**Figure 4:**
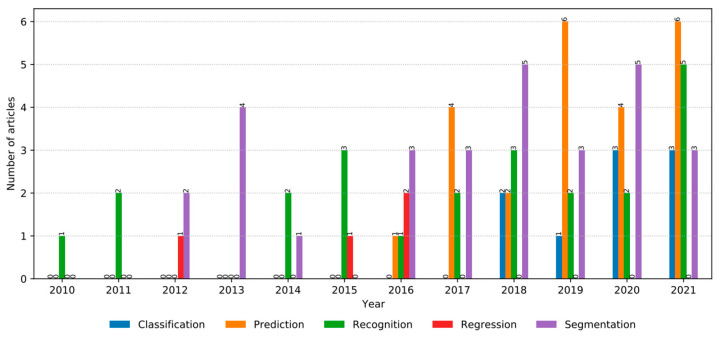
Distribution of AI/ML Problem types.

Several different AI problems and application methods were used for disease management purposes. The following graph, seen in Figure 4, depicts the distribution of AI/ML problem types in all the articles. This graph shows that the most prevalent AI problem type used for Inherited Retinal Disease is the Segmentation method, with a percentage of 33%. Regarding the meta-analysis results shown in Table 1, most studies included classification with a 50% rate. There are many definitions of these AI problems, and to clarify, the following applies. Here, classification refers to image classification, which identifies and labels groupings of pixels or vectors inside an image based on specific criteria that can be supervised or unsupervised [41]. As for a prediction, or forecasting, we refer to prediction models as part of machine learning, which projects the specific outcome values of a given input [42]. Recognition, in other words, identification, in this context, refers to object recognition which is a computer vision technology used to recognize items in photos or movies [43]. The objective is to train a computer to do what people do naturally: to comprehend (recognize) what is contained in a picture by identifying individuals, objects, settings, and visual characteristics [43].

On the other hand, regression in the context of AI enables the identification of intricate relationships involving input data and the automated recognition of correlations with inputs and outputs [44]. A DL technique that links a label or category with each pixel in an image is known as semantic segmentation. The segmentation AI problem refers to this and is used to identify a group of pixels that fall into separate categories [45].

**Figure 5:**
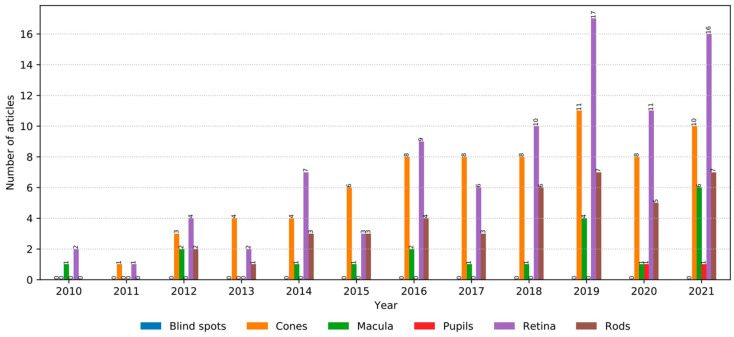
Distribution of eye parts affected by IRD.

In this study, the affected eye parts were also considered and identified as the following: blind spots, cones, macula, pupil, retina, and rods. The resulting distribution can be seen in the following Figure 5. The majority was related to the retina with a 40% occurrence rate, followed by cones with a 32% occurrence rate.

## 5. Conclusions

This systematic review examined artificial intelligence strategies used in managing Inherited Retinal Diseases, from diagnosis through therapy. In total, 11 articles were further analyzed with meta-analyses. Given the inclusion and exclusion criteria, it can be reported that the number of studies focusing on the use of artificial intelligence methods in the field of IRD is scarce. Further development is needed in the area to aid physicians and perform informed medical decisions. We have identified the following five answers to the critical research questions that were explored in this study:

1. Which artificial intelligence, machine learning, and deep learning methods are used for IRD management? (RQ1)
  - Several techniques are used in IRD management, most of them relating to detecting the disease using retinal imaging modalities. These methods refer to one or more AI/ML/DL tasks such as classification, prediction, recognition, regression, and segmentation.

2. In which way can these methods improve IRD detection? (RQ2)
  - These methods can improve IRD detection in several ways. They help segment and classify Ellipsoid Zones to facilitate the detection of IRD types as the change in these can signify different diseases. For the cone structure, localization and detection are possible with AI’s help and can be used as markers for disease types such as Stargardt Disease or Blue Cone Monochromacy. AI methods also aid researchers to identify genetic markers for Macular Dystrophy induced by ABCA4 and RP1L1 gene mutations and Retinitis Pigmentosa caused by the EYS gene. These methods also can classify effectively between IRD types and improve the outcome of targeted therapies. With the use of gradient visualization techniques, the ways can also explain the decision of such classifications. This can aid physicians in differentiating between false predictions.

3. Which algorithms/architectures are most used in IRD management? (RQ3)
  - Various algorithms were employed for the AI/ML/DL tasks, including CNNs, MDRNNs, Ensemble Classifiers, DNNs, Feature Extraction, SVMs, and DCNNs. As per the models used, 12 distinct models were identified: MatConvNet, MDLSTM blocks, Random Forest, Inception V-3, ResNet-UNet, Linear SVM, RBF, ResNet101 VGG19, LeNet, Inception Resnet V2, and Xception.

4. What are the complications of using these methods for IRD detection? (RQ4)
  - The main issues identified with the application of AI methods for IRD detection were the scarcity of data, lack of exploitation of representative populations including rare IRD types, image noise, movement artifacts, lack of specified characteristics in the selection of images, shadows created by the image acquisition technique or diseases presenting themselves as another.

5. What imaging modalities are used along with these methods for different types of IRD identification? (RQ5)
  - The different imaging modalities used for IRD detection are Optical Coherence Tomography, Adaptive Optics Scanning Light Ophthalmoscope, Spectral Domain Optical Coherence Tomography, Fundus Autofluorescence, Pupillometer, and Fundus Photography. They vary from the application to a specific disease type and applied algorithms.

The study has identified that AI in IRD management can be highly beneficial to the field. It can help ophthalmologists make informed decisions regarding the specific IRD disease type identification and assist with selecting patient specialized medical treatments by effectively identifying the genotypes of IRD diseases. It can significantly reduce false predictions and false treatment options. With gradient mapping techniques, it is also possible to present the result of the decision process of AI and the contributors of the last categorization or segmentation methods. Ophthalmologists can benefit highly from these visualization results, and they should be accessible to them so they can make better-informed decisions rather than solely relying on the classification result. Combined with the emerging telemedicine approaches, such as handheld-fundus cameras, or other smart-phone based applications, it might even be possible to detect and classify the subtype of the IRD via the Internet of Things devices and analyze it remotely. This can change the course of access to medical practice and include more people worldwide, including in rural areas. It might even contribute to the distribution of the collected IRD database and possibly improve rare IRD types of representation. Thus, leading to better classification results.

Overall, it can significantly save a vast amount of time and effort and create a less chaotic working environment for healthcare workers. Thus, providing better patient care. The use of AI can also aid researchers in visualizing such diseases. The segmentation and localization methods assist the phenotyping process and better understand the disease’s development. When further investigated, the underlying cause of the disease progression can be correlated with other factors and may help prevent the disease or the progression. Although the adaptation of AI to the clinical field has presented valuable outcomes by reducing the amount of workload on healthcare workers, as well as improving patient care by providing patient-specific therapeutic solutions and overall lowering costs, it should be still considered not the only method of analysis but rather a consultation to the search for better care. There still exist ethical and safety concerns regarding the use of AI in such medical circumstances. More unified policies should be provided by the policymakers and respective guidelines.

All in all, there are various techniques discovered in the systematic review. The benefits and challenges of using such methods are identified, and their possible results. For the future direction, the utilization of more enormous datasets incorporating comprehensive IRD populations with even rare disease types is advised. The field can benefit highly from the collaboration of institutions and researchers to gather uniform datasets and test the latest applications of artificial intelligence techniques.

## Figures and Tables

**Table 1:** Study Analysis.

Reference	Disease Type (s)	Imaging Modality	Dataset Size	Patient Distr.	Purpose	AI Problem	Algorithm	Model (s)	Best Performance
Camino et.al. (2018) [28]	Chloridemia, Retinitis Pigmentosa (RP)	Optical Coherence Tomography (OCT)	20 OCT Scans	20 Chloridemia, and 22 RP Subjects	To develop an adaptable method for different retinal diseases, using a DL method with multi IRD training for segmentation of preserved Ellipsoid Zone (EZ).	Segmentation, Classification	CNNs	MatConvNet	JSS: 0.912 ± 0.055
Davidson et.al. (2018) [29]	Stargardt Disease (STGD)	Adaptive Optics Scanning Light Ophthalmoscope (AOSLO)	290 images	8 STGD, and 17 Healthy Subjects	To automatically detect cones in both healthy and unhealthy subjects with STGD using MDRNN from AOSLO images.	Segmentation, Classification	MDRNNs	MDLSTM blocks	Dice Score: 0.9577
Wang et.al. (2018) [30]	Chloridemia	Optical Coherence Tomography (OCT)	20 OCT Scans	9 Chloridemia, and 5 Healthy Subjects	To automatically detect continuous areas of preserved EZ structure in order to identify Chloridemia from OCT images with ML techniques.	Segmentation	Ensemble Classifiers	Random Forest	JSS: 0.876 ± 0.066
Fujinami-Yokokawa et.al. (2019) [31]	Macular Dystrophy, Retinitis Pigmentosa (RP)	Spectral Domain Optical Coherence Tomography (SD-OCT)	178 SD-OCT scans	30 Macular Dystrophy, 28 RP, and 17 Healthy Subjects	To predict genes responsible for IRD in Macular Dystrophy and compare with RP using DL methods.	Prediction, Classification	DNNs	Inception V-3	Accuracy: 1.0
Charng et.al. (2020) [32]	Stargardt Disease (STGD)	Fundus Autofluorescence (FAF)	47 images	24 STGD Subjects	To use hyperautofluorescent flecks in FAF images to measure structural outcome in STGD1 using a DL based fleck segmentation method.	Segmentation	CNNs	ResNet-UNet	Dice Score: 0.80
Iadanza et.al. (2020) [33]	Retinis Pigmentosa (RP)	Pupillometer	30 chromatic pupillometry data	28 RP, and 10 Healthy Subjects	To define effective protocols and systems for an early diagnosis and monitoring through CP.	Classification	Feature Extraction, SVM	Linear SVM, Gaussian radial basis function (RBF)	Accuracy: 0.846, Sensitivity: 0.937, Specificity: 0.786
Miere et.al. (2020) [34]	Retinitis Pigmentosa (RP), Best Disease (BD), Stargardt Disease (STGD)	Fundus Autofluorescence (FAF)	483 images	73 Healthy, and 125 STGD, 160 RP, 125 BD eyes	To automatically classify different IRDs such as STGD, RP, and BD by means of FAF images using a DL algorithm.	Classification	CNNs	ResNet101	ROC-AUC: 0.999 PRC-AUC: 0.999
Shah, Ledo, and Rittscher (2020) [35]	Stargardt Disease (STGD)	Optical Coherence Tomography (OCT)	749 OCT scans	60 STGD, and 33 Healthy Subjects	To identify whether DL might be utilized for the automated classification of OCT images from patients with STGD using a smaller dataset.	Classification	CNNs	VGG19, custom LeNet	Accuracy 0.990, Sensitivity 0.998, Specificity 0.980 and JSS 0.990;
Sumaroka et.al. (2020) [36]	Blue Cone Monochromacy (BCM)	Optical Coherence Tomography (OCT)	42 OCT scans	26 IRD Subjects, 16 BCM Subjects	To predict the foveal visual outcomes of BCM treatment with different genotypes by using ML techniques on OCT images.	Prediction, Segmentation	Ensemble Classifiers	Random Forest	RSME: 0.159
Chen et.al. (2021) [37]	Retinitis Pigmentosa (RP)	Fundus Photography	1670 images	1153 RP, and 517 Healthy eyes	To detect the presence of RP based on color fundus photographs using a DL model.	Recognition, Classification	CNNs	Inception V3, Inception Resnet V2, and Xception	Accuracy: 0.960, AUROC: 0.9946, Sensitivity: 0.9571 Specificity: 0.9853 F3: 0.9599
Miere et.al. (2021) [38]	Geographic Atrophy (GA), Stargardt Disease (STGD), Pseudo-Stargardt Pattern Dystrophy (PSPD)	Fundus Autofluorescence (FAF)	314 images	110 GA, 204 STGD or PSPD eyes	To automatically classify GA on FAF images according to its etiology using DL techniques.	Classification	DCNNs	ResNet101	Accuracy: 0.921, AUC-ROC: 0.990

## Data Availability

Not applicable.
